# Serogroup X in Meningococcal Disease, Western Kenya

**DOI:** 10.3201/eid1306.070042

**Published:** 2007-06

**Authors:** Sadiki Materu, Helen S. Cox, Petros Isaakidis, Bienvenu Baruani, Thomas Ogaro, Dominique A. Caugant

**Affiliations:** * African Medical and Research Foundation, Nairobi, Kenya; † Médecins Sans Frontières–Spain, Nairobi, Kenya; Ministry of Health, Nairobi, Kenya; § Norwegian Institute of Public Health, Oslo, Norway

**Keywords:** Meningitis, serogroup X, outbreak, letter

**To the Editor:** Although >12 different serogroups of *Neisseria meningitidis* exist, most disease outbreaks across the African meningitis epidemic belt are caused by serogroup A and, less frequently, by serogroups C and W135 (*1*). *N. meningitidis* serogroup X was first described in the 1960s and has been found to cause a few cases of invasive disease across North America, Europe, and Africa (*2*). In Africa, small serogroup X outbreaks have been described in Ghana (9 cases over a 2-year period) and in Niger (134 cases between 1995 and 2000) (*3*,*4*). In 2006, however, 51% of 1,139 confirmed cases of meningococcal meningitis in Niger were found to be caused by serogroup X (*5*). Before the 2005-06 meningococcal epidemic season, no published reports had described serogroup X isolates in East Africa. We report the involvement of *N. meningitidis* serogroup X in an outbreak of meningococcal disease in Western Kenya.

In January 2006, the Ministry of Health of Kenya and Médecins sans Frontières were notified of a suspected meningococcal disease outbreak in West Pokot District, bordering Uganda, in Western Kenya. On the basis of the initial outbreak investigation, the outbreak was assessed to have begun in late December 2005. Subsequent active surveillance, using the same clinical case definition of sudden fever onset with stiff neck, altered mental status, or both, showed 74 suspected cases through mid-March 2006, with a case-fatality rate of 20%. No cases were reported after March 2006.

Over the course of the outbreak, cerebrospinal fluid samples were obtained from 18 patients. Due to low population density, poor access to seminomadic populations, and the limited nature of the outbreak (relatively small numbers dispersed over a wide geographic region), obtaining specimens from untreated patients in West Pokot proved difficult. Three of the 5 first samples were found to show gram-negative diplococci on staining, the next 2 were negative on Pastorex rapid latex agglutination test (Bio-Rad Laboratories, Hercules, CA, USA) (during the outbreak investigation), and a subsequent 13 were sent to the African Medical and Research Foundation (AMREF) laboratory in Nairobi, Kenya, for culture and susceptibility testing. From these 13 specimens, 2 yielded a pure growth of *N. meningitidis* serogroup X, while no growth was observed for the remaining 11 specimens. These 2 cultures were subsequently confirmed as serogroup X by the World Health Organization Collaborating Centre for Meningococci in Oslo, Norway. Multilocus sequence typing and sequencing of the *porA* and *fetA* genes as described (http://pubmlst.org/neisseria/), showed that the infecting strain belonged to a new sequence type, ST-5403, and that it was subtype P1.19,26 and FetA type F3-27. This sequence type is unrelated to other serogroup X isolates from Africa, including those from the latest serogroup X outbreak in Niger, but it resembles a sequence type isolated in the United States in the 1970s. In addition to the testing at AMREF and in the Oslo laboratory, the 13 samples were also analyzed by PCR at the US Naval Medical Research Unit No. 3 in Cairo, Egypt. Overall, 5 of these 13 specimens were positive for serogroup X (including the 2 samples found to be serogroup X at AMREF and confirmed by PCR in Oslo) and 1 each was positive for serogroups C, W135, and Y.

At the same time as this outbreak in Western Kenya, a meningococcal meningitis outbreak was being monitored across the border in the Karamoja region of northeastern Uganda. Seminomadic populations move freely across the 2 countries, and we can assume that there was 1 meningitis outbreak that started in eastern Uganda and spread to Western Kenya. Although initial laboratory testing in Uganda suggested the presence of serogroup A, among 23 specimens subsequently sent to the Oslo laboratory, 11 were identified as serogroup X by PCR and 3 were serogroup W135 (*6*). Therefore, the outbreaks in both Kenya and Uganda involved multiple *N. meningitidis* serogroups. In West Pokot, Kenya, the Ministry of Health and Médecins sans Frontières conducted a vaccination campaign using the trivalent polysaccharide vaccine against serogroups A, C, and W135.

Before 2006, previous disease outbreaks caused by serogroup X had not reached the magnitude of those caused by serogroups A, C, or W135; they tended to evolve independently of the occurrence of both serogroups A and C and to be self-limited (*3*,*4*). Although most of Kenya is not included in the African meningitis belt, large epidemics of meningococcal disease have been reported previously (*7*). In conclusion, we would like to highlight the presence of *N. meningitidis* serogroup X in East Africa, its potential involvement in disease outbreaks, and the difficulties it may cause for laboratory confirmation and, consequently, for making an appropriate epidemic response.
